# Enhancing clinically cardiovascular machine learning model for risk prediction via sample augmentation

**DOI:** 10.3389/fmed.2026.1849464

**Published:** 2026-06-09

**Authors:** Xiaoyu Tang, Min Tang, Wu Liu, Shaoyang Cui

**Affiliations:** 1 Shenzhen Hospital (Fu Tian) of Guangzhou University of Chinese Medicine, The Sixth Clinical School, Guangzhou University of Chinese Medicine, Shenzhen, Guangdong, China; 2 The Sixth Clinical Medical School, Guangzhou University of Chinese Medicine, Guangzhou, Guangdong, China; 3 South China Research Center for Acupuncture and Moxibustion, Medical College of Acu-Moxi and Rehabilitation, Guangzhou University of Chinese Medicine, Guangzhou, Guangdong, China; 4 Department of Chemistry, National University of Singapore, Singapore, Singapore; 5 College of Acupuncture and Orthopedics, Hubei University of Chinese Medicine, Wuhan, Hubei, China

**Keywords:** cardiovascular risk, machine learning, random forest, risk prediction, sample augmentation, SHAP

## Abstract

**Background:**

Small sample dataset and heterogeneous distributions limit the robustness and implementability of machine learning models for structured clinical data. To evaluate the value of moderate data augmentation for cardiovascular risk modeling and propose an interpretable and deployable solution within a “continuous risk to thresholding” framework.

**Methods:**

The heart disease classification dataset was randomly divided into training and validation sets with an 8:2 ratio. Constrained feature space augmentation was performed within the training set, and the effects of four thresholds (0×, 1×, 2×, and 3×) on support vector regression (SVR), random forest (RF), extreme gradient boosting (XGBoost), light Gradient Boosting Machine (LightGBM), and multi-layer perceptron (MLP) were compared. Continuous risk scores were evaluated using mean absolute error (MAE), root mean squared error (RMSE), and R^2^. The output confusion matrix was then thresholded. Interpretative analysis was performed using SHapley Additive exPlanations (SHAP), and partial dependence plot (PDP).

**Results:**

2 × augmentation achieved the favorable compromise between error (reduced MAE and RMSE) and goodness of fit (increased R^2^). Under the 2 × condition, RF performed favorable overall, achieving an accuracy of 94.0%, an F2 of 94.4%, a sensitivity of 95.9%, and a specificity of 91.8% after thresholding. SHAP/PDP shows that oldpeak, num major vessels, chest pain type, thal, exang, and max hr. are the primary driving factors, exhibiting stable patterns such as monotonic, step, and threshold. Moderate data augmentation (preferably 2×) can significantly improve robustness in small sample settings; RF achieves the favorable balance between accuracy, stability, and interpretability.

**Conclusion:**

Combining SHAP/PDP’s multi-layered interpretation and thresholding approach, this study provides a reusable multiplication guidance and risk stratification scheme, providing a methodological basis for deploying interpretable cardiovascular risk models.

## 1 Introduction

Cardiovascular disease remains one of the leading causes of death and disability worldwide. Even in the era of modern medicine and drug treatment, early identification of high-risk individuals remains key to improving outcomes (1–3). The linear weighted risk scores commonly used in clinical practice have limitations in dealing with population heterogeneity, strong nonlinear relationships, and multivariate interactions, and are difficult to meet the threshold requirements for screening, triage, and interventional assessment in different clinical scenarios (4, 5). Therefore, there is a clear clinical and methodological necessity to build predictive tools that are interpretable, reusable, and threshold-sensitive (6, 7).

In recent years, machine learning (ML) has been studied for clinical diagnosis and early risk identification (8–13). It can capture nonlinearities and high-order interactions from multidimensional heterogeneous data, output quantifiable, updateable, and scenario-based risk scores, and then connect with clinical actions such as triage, screening, review frequency, and intervention intensity to promote earlier, more precise, and personalized decision support (14–16). In recent years, research on cardiovascular disease has shown that it outperforms traditional linear scores in event prediction, readmission/death risk stratification, and treatment response assessment. Tree models (such as RF and gradient boosting) and small neural networks are particularly effective on structured data, and combined with methods such as SHapley Additive exPlanations (SHAP)/partial dependence plot (PDP), an interpretable risk factor profile has been initially constructed (17–19). However, there is still a key bottleneck in translating these advances into bedside value: model training and accuracy issues caused by sample data. The lack of sample data has led to “a good cook cannot cook without rice”, which has seriously restricted the development and application of machine learning in clinical diagnosis and risk identification (20, 21).

To alleviate the high variance and instability caused by the small sample size and sparse distribution of clinical structured data, various strategies have been proposed in the literature, including regularization/feature selection, ensemble learning, transfer/semi-supervised learning, and data augmentation (22, 23). Compared with the model side, sample amplification directly increases the effective density on the data side and fills the gaps in the key decision boundaries. It can reduce variance and overfitting, stabilize cross-validation and extrapolation performance, alleviate extreme fluctuations during category imbalance and thresholding, and be universally compatible with multiple algorithms without changing the model structure. However, the key to the sample amplification method is to perform local interpolation/mild perturbations within the clinically reasonable value domain and similar neighbors to avoid noise extrapolation. It is important to compare different amplification multiples, monitor distribution drift and performance decline, and find the optimal degree.

Based on the above ideas, we propose to construct a feature-space constrained sample augmentation method on a cardiovascular structured dataset. Within the training set, we perform a moving average local synthesis based on similar neighbors and superimpose small Gaussian perturbations. We systematically compare the effects of four augmentations (0×/1×/2×/3×) on support vector regression (SVR), random forest (RF), extreme gradient boosting (XGBoost), light Gradient Boosting Machine (LightGBM), and multi-layer perceptron (MLP). We evaluate the quality of fit of continuous risk scores using mean absolute error (MAE)/root mean squared error (RMSE)/R^2^, and threshold the output to generate a confusion matrix for clinical judgment. Combining Permutation/Gini-SHAP-PDP, we form a consistent chain of evidence from global to individual to marginal effects. By providing reusable multiplication guidance and thresholding paths, this study aims to provide a practical methodological solution for small-sample clinical modeling, alleviating performance issues caused by sample scarcity.

## 2 Materials and methods

### 2.1 Data

This study used the Heart Disease Classification dataset. The data used contained the following 13 features: age, sex, chest pain type (cp), resting bp (trestbps), cholesterol (chol), fasting blood sugar (fbs), restecg, max hr. (thalach), exang, oldpeak, slope, num major vessels (ca), thal, as well as a binary target. Continuous variables included: age, resting bp, cholesterol, max hr., and oldpeak. Categorical variables included: sex, chest pain type, fasting blood sugar, restecg, exang, slope, num major vessels, and thal. Target value included: target (0/1). For this study, the dataset was randomly split into training and validation sets in an 8:2 ratio using a fixed random seed.

### 2.2 Data augmentation

Use the moving average method to amplify and enhance the original dataset (24, 25). The basic idea of this method is to calculate the average value of data points containing a certain number of items based on time series data, thereby achieving long-term prediction of data trends. In addition, the moving average method can eliminate random fluctuations in the prediction and more accurately analyze and predict the direction and trend of water quality changes. The basic principles of using this method for data amplification are summarized as follows. For a specific measurement dataset, the following formula is used (Equation 1).

$$\bar{x_{\mathrm{ti}}}=\frac{x_{t}+x_{t+1}+\ldots +x_{t+i-1}}{i}(1\leq t<n)$$ (1)

Where t represents an item in the dataset augmentation, $\bar{x_{\mathrm{ti}}}$ represents the amplified data, and i represents the number of data points included in the moving average. When the number of items is 2, 3, or 4, the corresponding augmented dataset is $S_{2}=(\bar{x_{12}},\bar{x_{22}},\ldots ,\bar{x_{(n-1)2}})$ , total n-1 items. $S_{3}=(\bar{x_{13}},\bar{x_{23}},\ldots ,\bar{x_{(n-2)3}})$ , total n-2 items. $S_{4}=(\bar{x_{14}},\bar{x_{24}},\ldots ,\bar{x_{(n-3)4}})$ , total n-3 items. In order to compare the prediction performance differences of different amplification factor models in the modeling process, predictive modeling analysis was performed on four datasets, including the original dataset.

### 2.3 Model

**Figure 1:**
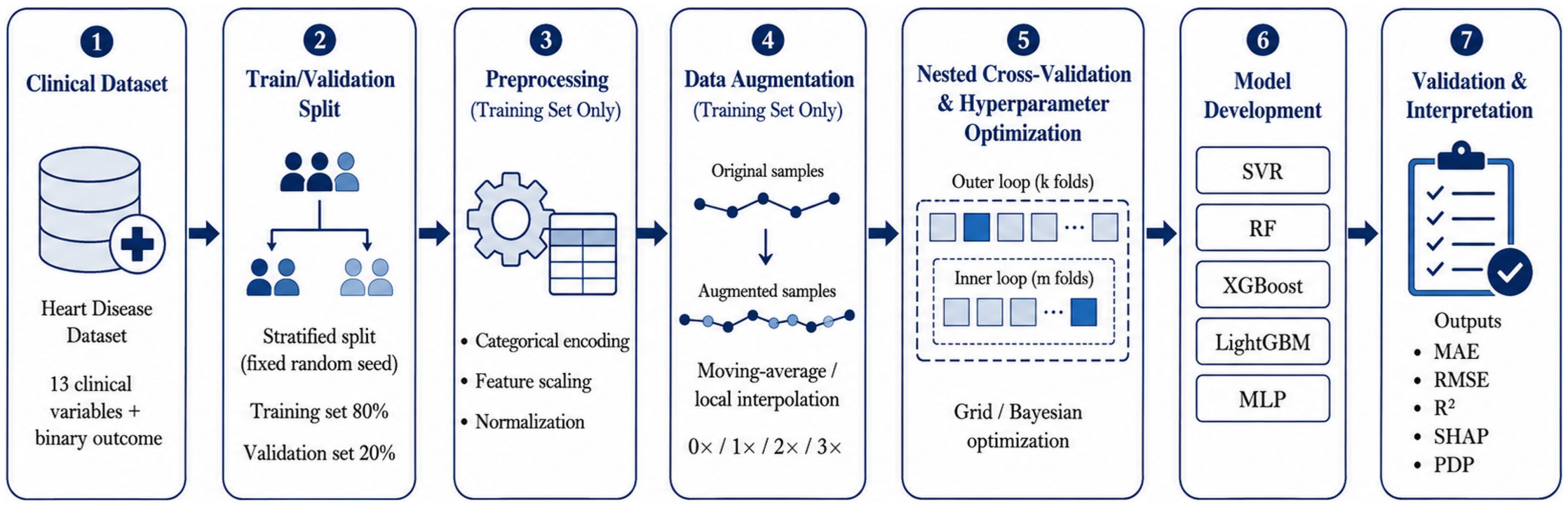
Simplified workflow of the augmented machine learning framework for heart disease prediction. The pipeline proceeds from dataset acquisition and train/validation splitting, through training-set-only preprocessing and data augmentation, to nested cross-validation with hyperparameter optimization, model training, and final validation and interpretation.

Univariate and multivariate logistic regression analysis was used to identify predictors. Using training data, five machine learning models were developed: SVR, RF, XGBoost, LightGBM, and MLP. The prediction accuracy of the model is evaluated using a validation set. The overall modeling workflow is illustrated in Figure 1. Dataset splitting, preprocessing, and augmentation were performed in sequence, with all operations applied exclusively to the training set. Nested cross-validation and hyperparameter optimization were then conducted within the training set before evaluating performance on the independent validation set. This workflow ensures a rigorous, leakage-free pipeline and facilitates reproducibility. Data augmentation was conducted using a moving-average-based local interpolation strategy across neighboring samples, with multiple augmentation levels (0×, 1×, 2×, 3×) explored. The augmentation was constrained within the feature space of clinically plausible values to minimize the generation of unrealistic or biased synthetic samples. Although a full comparison with standard oversampling techniques such as SMOTE was not conducted in this study, future work will include benchmarking against SMOTE to further validate the augmentation strategy. To avoid potential data leakage, dataset splitting was performed prior to preprocessing and augmentation procedures. All interpolation, scaling/encoding, and data augmentation operations were conducted exclusively within the training set, while the validation set remained completely independent throughout model training and evaluation. Preprocessing steps, including categorical encoding, feature scaling, and normalization, were applied sequentially within the training set, and nested cross-validation was conducted with an inner loop for hyperparameter optimization and an outer loop for model evaluation, as illustrated in Figure 1. Model selection and early stop are used using nested cross-validation (or 60/20/20 allocated internal CV). Hyperparameters were determined using grid/Bayesian optimization based on validation-set performance (MAE, RMSE, and R^2^), and detailed model configurations are provided in Supplementary Table 1. To improve reproducibility, model training, validation splitting, and optimization procedures were conducted using fixed random seeds where applicable. Feature importance of tree-based models was further interpreted using SHAP and PDP analyses based on established interpretable machine learning frameworks for clinical risk prediction (26, 27). When the model output is used for probability interpretation, probability calibration is performed using Platt or equivalent regression. Model outputs were interpreted as continuous cardiovascular risk scores before thresholding. Accordingly, MAE, RMSE, and R^2^ were retained as auxiliary metrics to describe the numerical consistency between predicted risk scores and the true binary outcomes at the risk-score level. Classification performance was primarily evaluated using ROC-AUC, PR-AUC, calibration curves, Brier score, and thresholded classification metrics, including accuracy, sensitivity, specificity, PPV, NPV, F1-score, and F2-score. The binary classification threshold was selected using the Youden index to balance sensitivity and specificity. Cross-validation variability was reported as mean ± standard deviation for MAE, RMSE, and R^2^ across different augmentation factors and models. Training and inference resource consumption is also recorded for deploymentability assessment.

The coefficient of determination (R^2^), root mean square error (RMSE), and mean absolute error (MAE) were used as auxiliary metrics to evaluate the numerical agreement between continuous predicted risk scores and the true binary outcomes at the risk-score level (Equations 2–4). Standard classification-oriented metrics, including ROC-AUC, PR-AUC, calibration analysis, Brier score, and thresholded classification metrics, were further used to evaluate discrimination, probability calibration, and clinical classification performance.

$$R^{2}=1-\frac{\sum_{i=1}^{n}{\left(\hat{Y_{i}}-\bar{Y}\right)}^{2}}{\sum_{i=1}^{n}{\left(Y_{i}-\bar{Y}\right)}^{2}}$$ (2)

$$\text{RMSE}=\sqrt{\frac{1}{n}\sum_{i=1}^{n}{\left(\hat{Y_{i}}-Y_{i}\right)}^{2}}$$ (3)

$$\mathrm{MAE}=\frac{1}{n}\sum_{i=1}^{n}|\hat{Y_{i}}-Y_{i}|$$ (4)

Generally speaking, R^2^ is the ratio of the variance of the overall data and the degree of correlation in the prediction process of the observation model, and RMSE and MAE are error indicators presented in the same unit as the target variable.

The Non-dominated Sorting Genetic algorithm II (NSGA-II) was improved from the NSGA, which is one of the most popular multi-objective genetic algorithms (28, 29). The NSGA-II has the advantages of fast computation and good convergence of the solution set and can optimize multiple objectives and provide a set of Pareto solutions (29). In the present study, the objective function was characterized by the output function of the regression modeling with the favorable predictive performance (30).

## 3 Results

### 3.1 Data feature distribution

**Figure 2:**
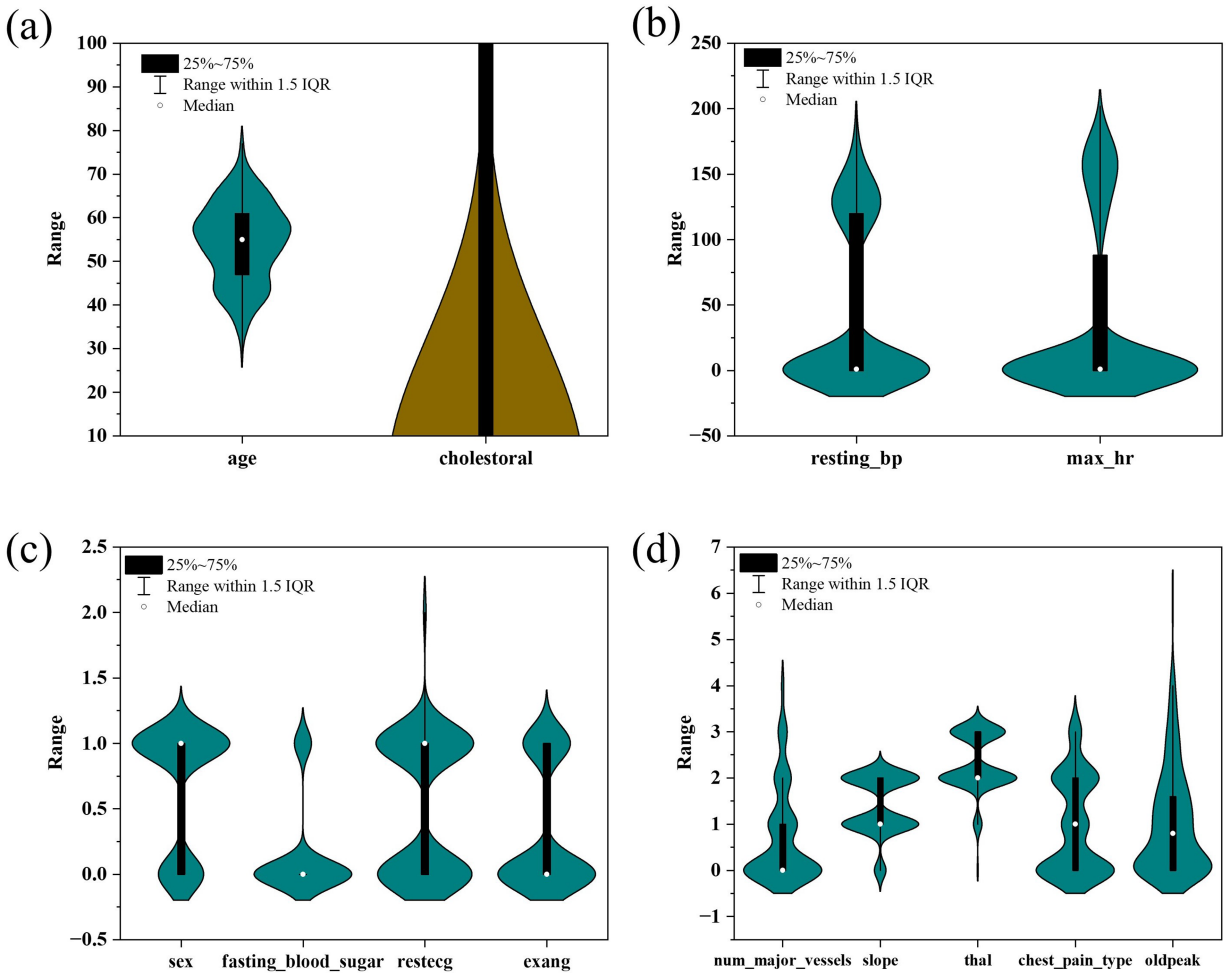
Distribution of the 13 features of the original dataset. **(a)** Boxplot of age (age) and cholesterol (cholesterol); **(b)** Boxplot of resting blood pressure (resting_bp) and maximum heart rate (max_hr); **(c)** Distribution of sex (sex), fasting blood glucose (fasting_blood_sugar), resting electrocardiogram (restecg), and exercise-induced angina (exang); **(d)** Distribution of number of major vessels (num_major_vessels), ST-segment slope (slope), thalassemia (thal), chest pain type (chest_pain_type), and ST-segment depression (oldpeak).

The overall distribution of continuous variables is shown in Figures 2a,b. The median age was 55.0 years (IQR 13.5), with a nearly symmetric distribution (skewness −0.20) and an IQR-based outlier rate of 0.0%. The median resting bp was 130.0 mmHg (IQR 20.0), with mild right skewness (skewness 0.71) and an outlier rate of 3.0%. The median cholestoral value was 240.0 mg/dL (IQR 63.5), with significant right skewness (skewness 1.14) and a low proportion of extremely high values (outlier rate 1.7%). The median max hr. was 153.0 beats/min (IQR 32.5), with mild left skewness (skewness −0.54) and an outlier rate of 0.3%. The median oldpeak value was 0.8 (IQR 1.6), with significant right skewness (skewness 1.27) and an outlier rate of 1.7%. Overall, the skewness of continuous variables is mainly concentrated in the high-value areas of cholestoral and oldpeak, suggesting that nonlinear marginal effects may exist in high-quantile segments.

The distribution of categorical variables is shown in Figures 2c,d. For sex, the code “1” accounted for 68.3% (“0” accounted for 31.7%); for fasting blood sugar, the code “1” accounted for 14.9%; for restecg, the code “1” accounted for 50.2%, “0” accounted for 48.5%, and “2” accounted for 1.3%; for exang, the code “1” accounted for 32.7%. Among the structure/function-related variables, num major vessels: coded “0” accounted for 57.8%, “1” accounted for 21.5%, “2” accounted for 12.5%, “3” accounted for 6.6%, and “4” accounted for 1.7%; slope: coded “2” accounted for 46.9%, “1” accounted for 46.2%, and “0” accounted for 6.9%; thal: coded “2” accounted for 54.8%, “3” accounted for 38.6%, “1” accounted for 5.9%, and “0” accounted for 0.7%; and chest pain type: coded “0” accounted for 47.2%, “2” accounted for 28.7%, “1” accounted for 16.5%, and “3” accounted for 7.6%. While the distributions above indicate that most variables are not extremely skewed, there are significant structural differences in the category proportions of num major vessels, slope, thal, and chest pain type, providing objective context for subsequent feature coding and PDP and SHAP interpretation.

### 3.2 Correlations and principal component structure

**Figure 3:**
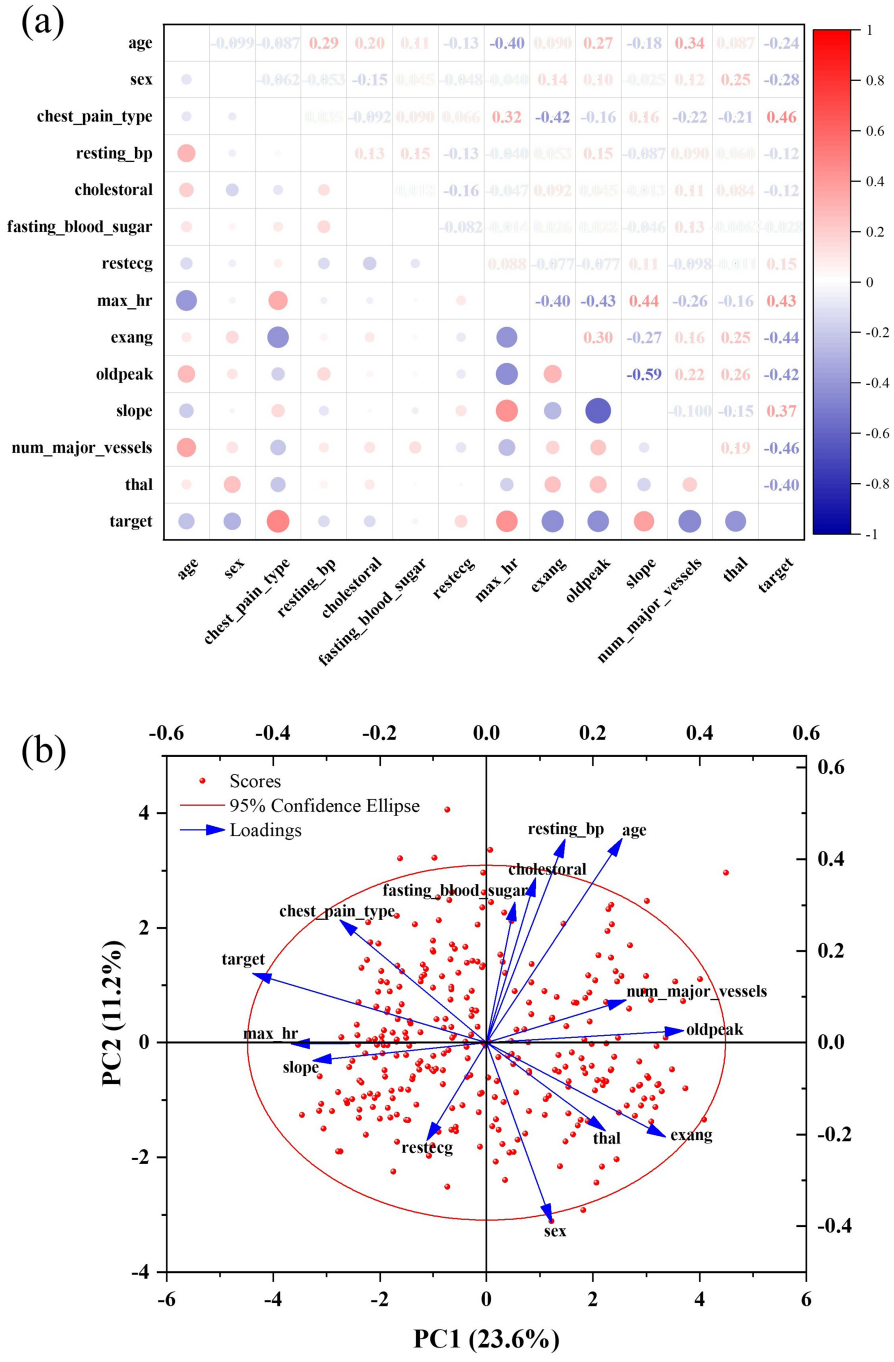
Feature correlations and principal components: **(a)** Pearson correlation heatmap; **(b)** PCA biplot (first two principal components). Ellipses represent 95% confidence intervals.

Among the continuous features, age correlated moderately and negatively with max hr. (*r* = −0.40) and weakly and positively with resting bp (*r* = 0.29) and cholestoral (*r* = 0.20); the association between resting bp and oldpeak was weak (*r* = 0.15). The remaining continuous–continuous pairs mostly had |r| < 0.20, indicating that multicollinearity was generally manageable. Feature–outcome associations were stronger for a small subset: chest pain type (*r* = 0.46), slope (*r* = 0.37), oldpeak (*r* = −0.42), max hr. (*r* = 0.43), and thal/num major vessels (|*r*| = −0.40 ~ −0.46) showed the largest absolute correlations with the target, in directions consistent with the SHAP/PDP findings. Results are based on Pearson’s r (n = 303); effect sizes are interpreted as weak (<0.30) or moderate (0.30–0.50). The remaining continuous variables were mostly weakly correlated (Figure 3a), suggesting that multicollinearity was generally manageable.

After principal component analysis (PCA) was performed on dummy variables for both continuous and categorical features, the first two principal components explained 16.5% (PC1) and 11.9% (PC2), respectively, for a total of 28.4%. The loading on PC1 was primarily driven by variables such as chest pain type levels, max hr., and oldpeak (with the highest absolute loadings), reflecting the “symptom-provoking-heart rate/ischemic burden” dimension. PC2 was significantly influenced by sex, restecg, age, and cholestoral (Figure 3b). Within the PC1–PC2 space, the 95% confidence ellipses for the two target categories were partially separated but still overlapped. The difference in center was significant along PC1, but not along PC2. These findings suggest that part of the discriminative information may be reflected along the first principal component, which is primarily influenced by variables such as chest pain type, max hr., and oldpeak and may reflect variation associated with exercise-related cardiovascular responses and ischemic characteristics within the current dataset. However, this interpretation should be regarded as exploratory rather than definitive, because PCA provides a statistical projection rather than direct physiological validation. The overlap between the two target categories also indicates limited linear separability within the current feature space, particularly given the skewed and stratified distributions of several clinical variables.

### 3.3 Different amplification factors and model performance

**Figure 4:**
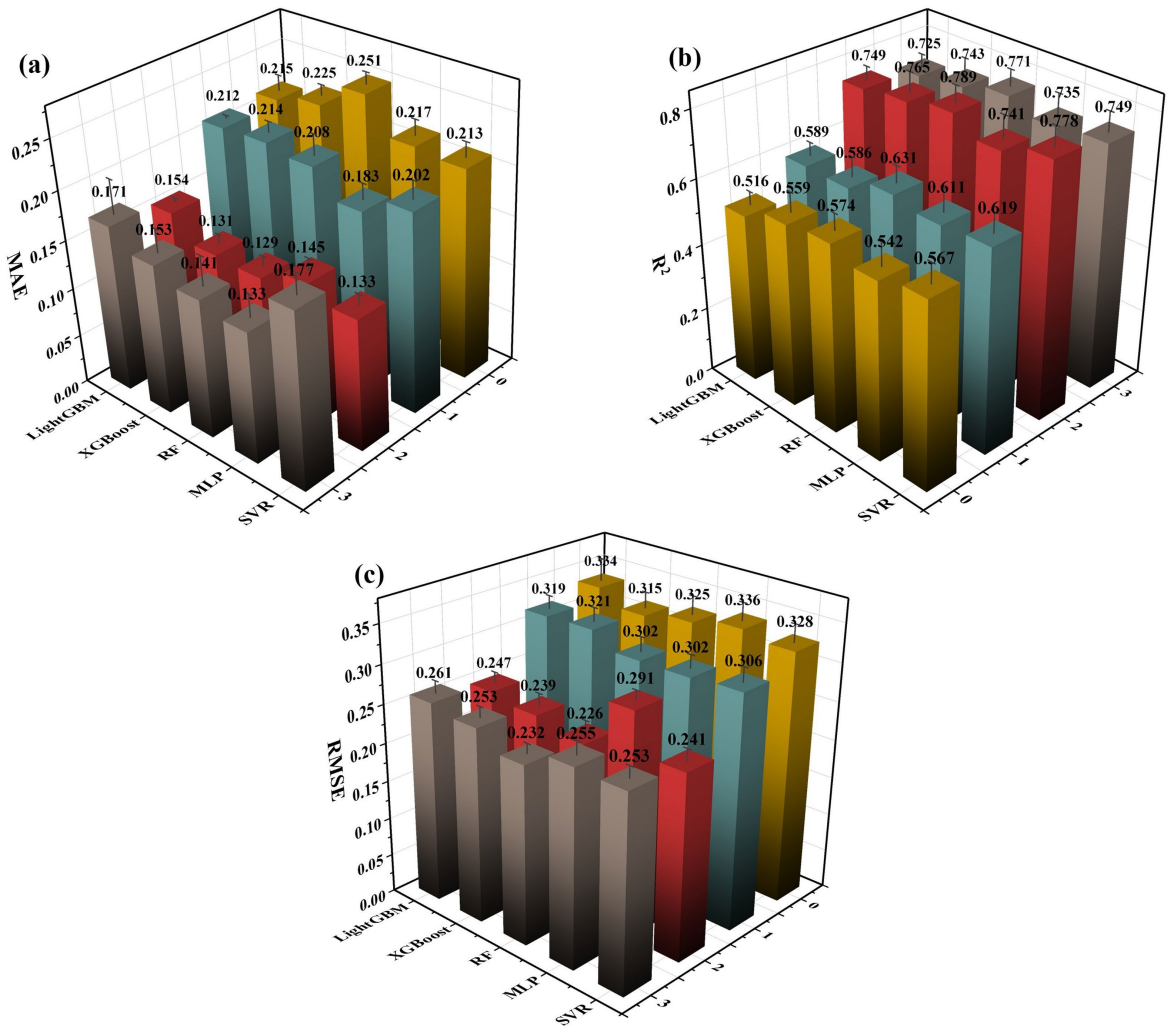
Performance comparison of SVR, RF, XGBoost, LightGBM, and MLP under different augmentation factors (0×, 1×, 2×, and 3×). Subplots show changes in **(a)** MAE, **(b)** R^2^, and **(C)** RMSE on the validation set. Moderate augmentation generally improved model performance, whereas excessive augmentation (3×) resulted in partial performance decline in several models.

A systematic comparison of five models, including SVR, RF, XGBoost, LightGBM, and MLP, was conducted under four scaling settings: 0×, 1×, 2×, and 3×. The three regression metrics showed consistent trends: from 0 × to 2×, MAE and RMSE decreased continuously, while R^2^ increased. When scaling to 3×, performance on the test set declined slightly (MAE/RMSE increased, R^2^ decreased), suggesting the generalization risk associated with overscaling (Figure 4). Among the evaluated models, RF achieved the most favorable overall balance at the 2 × augmentation level, exhibiting the lowest MAE/RMSE and the highest R^2^. In contrast, several models, particularly MLP and LightGBM, showed mild performance deterioration under the 3 × augmentation condition, suggesting potential over-smoothing or distributional distortion caused by excessive augmentation.

A horizontal comparison showed that RF achieved the favorable overall performance at 2 × scaling: the lowest MAE/RMSE, the highest R^2^, the lowest cross-fold variance, and the favorable stability. XGBoost and LightGBM performed next favorable, approaching RF at 2×, but becoming more sensitive to overscaling at 3×. SVR, constrained by the linearity assumption, exhibited relatively limited improvement. MLP performance improved with scaling, but experienced increased variance and a more pronounced decline in stability at 3 × (Figures 4a–c). To provide additional information on performance variability, MAE, RMSE, and R^2^ across different augmentation factors and models are reported as mean ± standard deviation in Supplementary Table 2.

### 3.4 SHAP interpretation analysis

**Figure 5:**
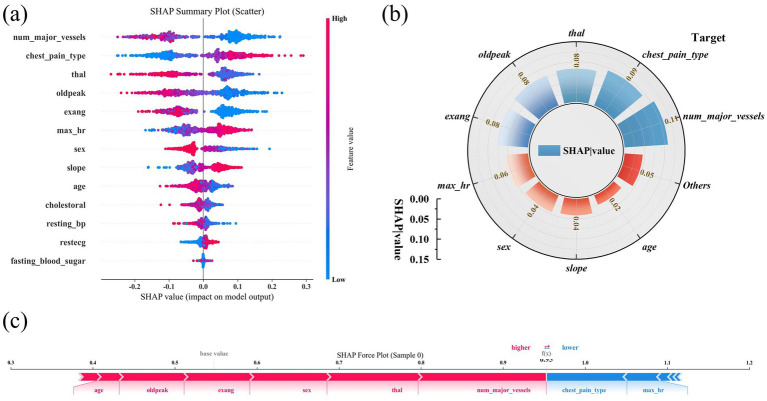
SHAP interpretation based on the RF model. **(a)** SHAP summary beeswarm plot; **(b)** Radial bar plot of feature importance for the random forest (RF) model; **(c)** Individual SHAP force plot, showing the feature contributions of typical high-risk and low-risk individuals.

Under a 2 × augmentation setting, global and individual interpretations of RF are consistent, indicating that the model relies primarily on a few key features (Figure 5). The SHAP summary beeswarm plot (Figure 5a) shows the top contributions in the order of num major vessels, chest pain type, thal, oldpeak, exang, max hr., sex, slope, and age. The contributions of the remaining variables (cholestoral, resting bp, restecg, and fasting blood sugar) are concentrated around zero. The point clouds for different features exhibit significant nonlinearity and heteroskedasticity. High values of oldpeak and exang correspond to clusters of positively skewed SHAP values, suggesting a consistent upward bias in risk scores. High values of max hr. are mostly in the negative SHAP range, suggesting a “protective” pattern. The point clouds for chest pain type, thal, and num major vessels exhibit a multimodal/stratified distribution, indicating significant differences in effects across coding levels, possibly accompanied by interactions. The overall impact of age on risk score increases with the value, but there is an overlapping band after superposition with other variables, suggesting nonlinear superposition.

The SHAP beeswarm plot (Figure 5a) illustrates the distribution, direction, and heterogeneity of feature contributions across individual samples, whereas the radial importance plot (Figure 5b) summarizes the aggregated global importance of each feature based on the overall SHAP magnitude. The consistency between the two visualizations further supports that the aforementioned top variables are the primary drivers of model predictions. The individual force plot (Figure 5c) illustrates the contribution breakdown for two representative individuals: in the high-risk individual, oldpeak, exang, sex, thal, and num major vessels collectively push the model output upward from the baseline, while chest pain type and max hr. counteract each other. In the low-risk individual, the opposite occurs, with negative contributions from max hr. and chest pain type dominating. This global-individual consistency provides a traceable description of model behavior for subsequent partial dependency analysis and thresholding decisions, rather than evidence of causal relationships between clinical variables and disease outcomes.

### 3.5 Partial dependence

**Figure 6:**
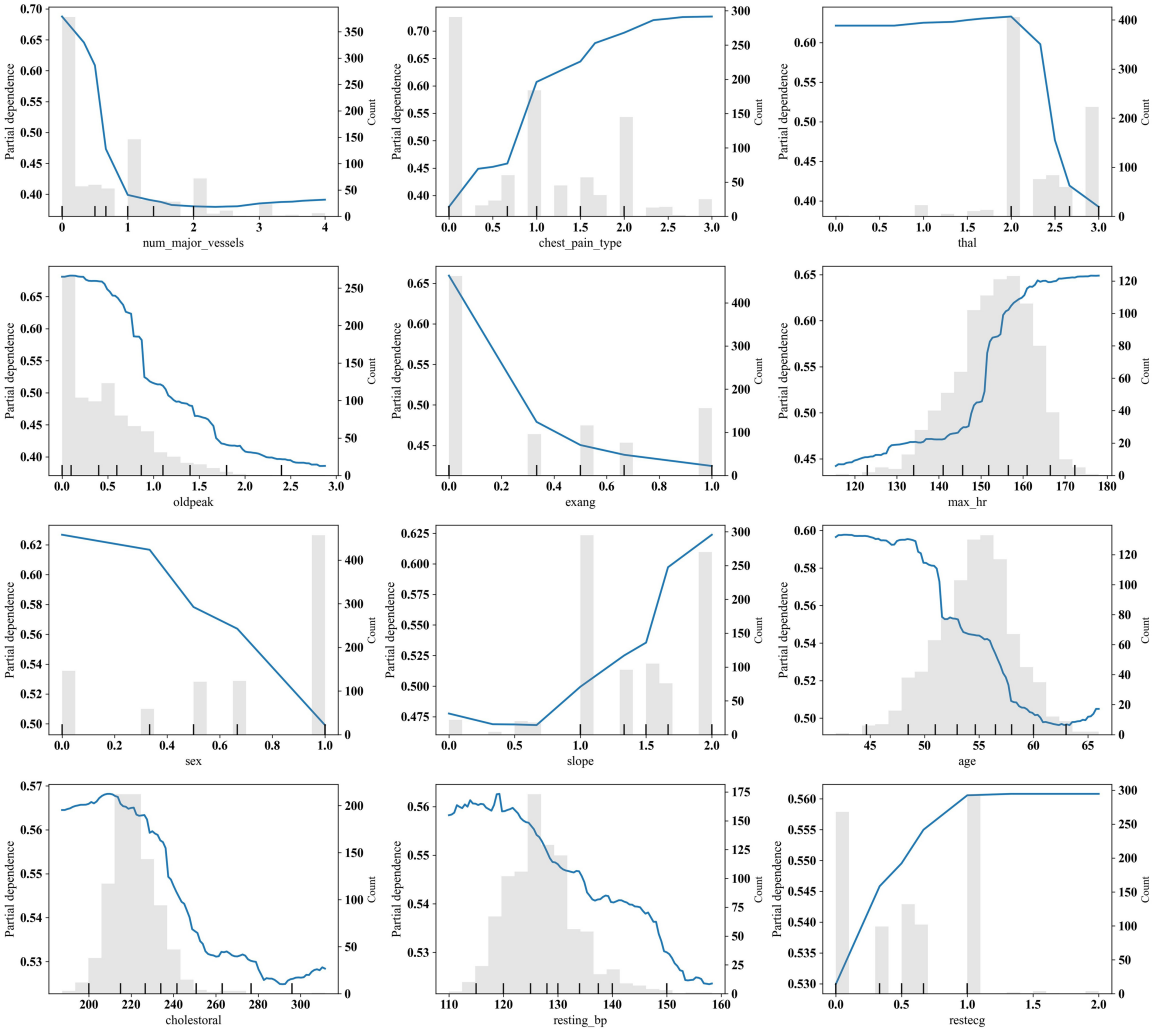
PDP of the top 12 contributing features.

Partial dependence plots (PDP) for the top 12 contributing features reveal clear monotonic or hierarchical relationships between several variables and the model output (Figure 6). For continuous variables, oldpeak exhibits a nearly monotonic positive relationship with the predicted value, with an increasing slope observed around 1.0–1.5, indicating a more pronounced upward push in the high-percentile range. Max hr. exhibits a negative correlation, transitioning from a slow decline to a plateau/slow decline around 140 beats/min, suggesting a stronger protective margin in the higher heart rate range. The curve for age rises slowly overall, becoming more convex after the middle-aged and elderly quantiles (approximately ≥55 years old). The marginal effects of cholesteral and resting bp are weak, with only a slight upward trend in the high tail, consistent with their lower SHAP rankings.

For categorical/ordinal variables, num major vessels shows a step-wise increase: higher coding levels correspond to progressively higher predicted scores compared to “0.” The curves for chest pain type and thal both show clear stratification (multimodal/segmented structure), with high coding levels pushing up the model output in most intervals. For exang, “1” produces a stable positive increment relative to “0.” Slope exhibits a nonmonotonic relationship, with the intermediate level exceeding the extremes in most intervals. The marginal effect of sex is positive and moderate. The curves for restecg and fasting blood sugar are generally close to horizontal, suggesting that their marginal contributions to the current model are limited. Overall, the PDP results are highly consistent with the aforementioned SHAP results: oldpeak, num major vessels, chest pain type, thal, exang, and max hr. are the primary drivers. Oldpeak and num major vessels exhibit a strongly monotonic/step-like pattern, while max hr. exhibits an inverse pattern. Chest pain type, thal, and slope exhibit stratification and nonlinearity. To mitigate potential bias in PDP due to feature correlation, we provide corresponding ICE/ALE curves in the Supplementary material to demonstrate robustness at both the individual and conditional expectation levels.

### 3.6 Discrimination, calibration, and thresholded classification performance

**Figure 7:**
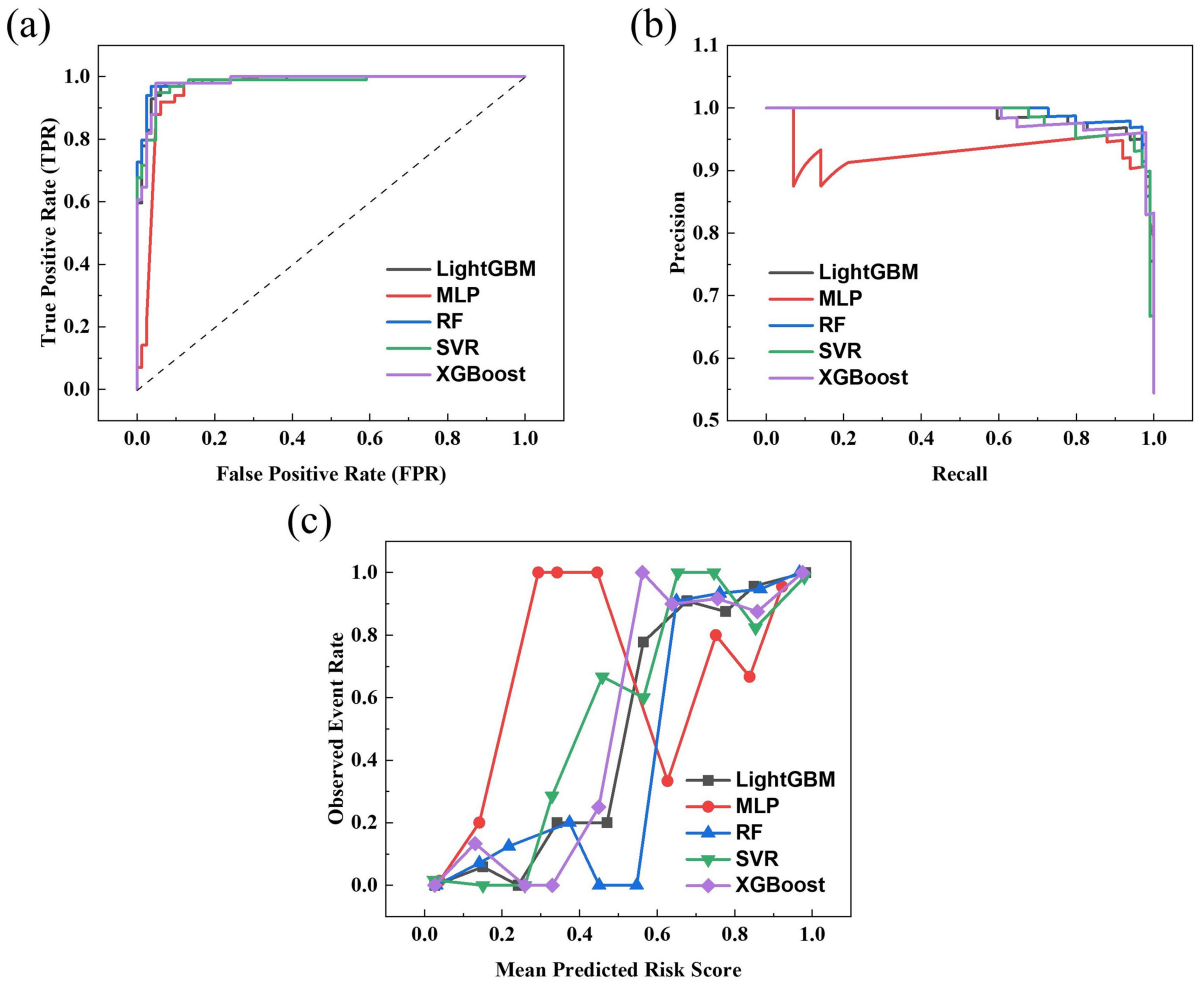
**(a)** ROC curves of the five evaluated models on the validation set. ROC-AUC was used to evaluate the discrimination ability of each model. **(b)** Precision-recall curves of the five evaluated models on the validation set. PR-AUC was used to assess model performance under class distribution imbalance. **(c)** Calibration curves of the five evaluated models on the validation set. The diagonal line represents ideal calibration, and Brier score was used to quantify calibration error.

To further evaluate the classification performance of the five models, ROC curves, precision-recall curves, and calibration curves were generated on the validation set. ROC-AUC was used to assess discrimination ability, as shown in Figure 7a. PR-AUC was used to evaluate model performance under class distribution imbalance, as shown in Figure 7b. Calibration curves, together with Brier score, were used to assess the reliability of predicted probabilities, as shown in Figure 7c. The threshold for binary classification was selected according to the Youden index, and thresholded classification metrics were summarized in Supplementary Table 3. Among the evaluated models, RF achieved the highest ROC-AUC (0.9887) and PR-AUC (0.9907), together with the lowest Brier score (0.0462), indicating a comparatively favorable balance among discrimination, precision-recall performance, and probability calibration. XGBoost showed very similar thresholded classification performance to RF, while LightGBM achieved relatively high specificity. Under the Youden index-based threshold, RF and XGBoost achieved the same accuracy of 96.15%, sensitivity of 96.97%, specificity of 95.18%, F1-score of 96.48%, and F2-score of 96.77%. Overall, RF showed the most favorable overall balance across ROC-AUC, PR-AUC, Brier score, and thresholded classification metrics.

**Figure 8:**
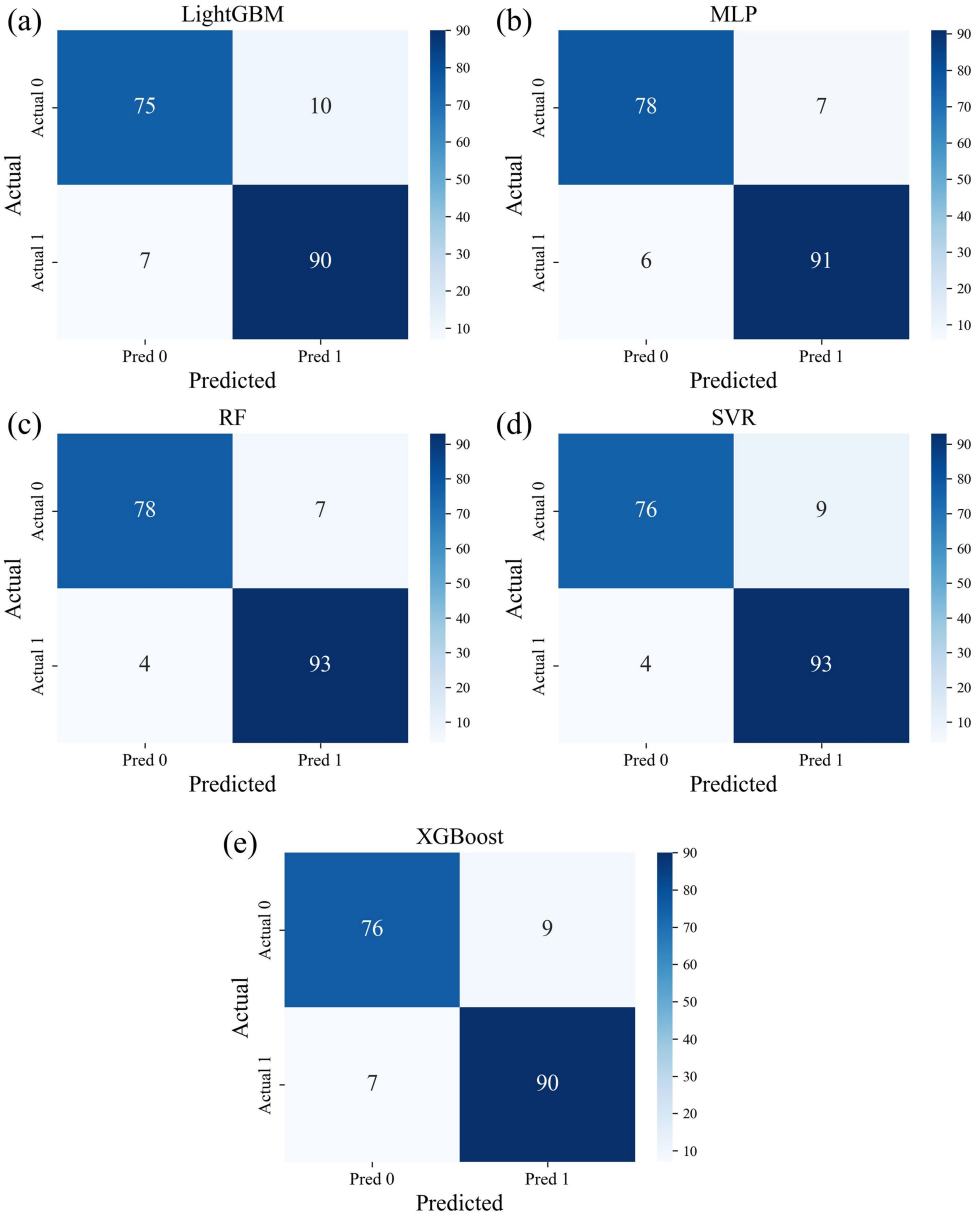
Prediction results of the confusion matrix of the five models. **(a)** LightGBM, **(b)** MLP, **(c)** RF, **(d)** SVR, **(e)** XGBoost.

At the recommended threshold, the confusion matrices for the five models are shown in Figure 8. The main discriminant metrics calculated from these models are as follows. RF and XGBoost achieved the highest thresholded classification performance under the Youden index-based threshold, with an accuracy of 96.15%, sensitivity of 96.97%, specificity of 95.18%, PPV of 96.00%, NPV of 96.34%, F1-score of 96.48%, and F2-score of 96.77%. Although their thresholded classification metrics were identical, RF further showed slightly higher ROC-AUC and PR-AUC and a lower Brier score than XGBoost, supporting its comparatively favorable overall performance. Its specificity of 91.8% and PPV of 93.0% remained high. MLP came in second (accuracy of 92.9%, F1 of 93.3%, sensitivity of 93.8%, and specificity of 91.8%), approaching RF overall but slightly lower in sensitivity and NPV. SVR tied with RF for the highest sensitivity (95.9%), but had a lower specificity (89.4%), resulting in slightly lower PPV (91.2%) and overall accuracy (92.9%). XGBoost and LightGBM performed similarly (accuracies of 91.2 and 90.7%, respectively). Their specificity (both ≤89%) and PPV (90.9 and 90.0%) were relatively low, suggesting a slightly conservative approach to negative class identification at the current threshold. Overall, RF provided the most balanced results in terms of the sensitivity-specificity and PPV-NPV trade-offs, consistent with the aforementioned regression metrics and interpretable analysis.

## 4 Discussion

### 4.1 Main findings

This study systematically evaluated the impact of a data augmentation strategy centered on “feature space moving average + restricted perturbation” on model performance. The results showed that 2 × augmentation achieved the favorable compromise between error (MAE/RMSE) and goodness of fit (R^2^); in multi-model comparisons, random forest (RF) ranked first in accuracy, F1, and sensitivity-specificity trade-offs. Explanatory analysis showed that the main discriminant information of the model was concentrated on variables such as the number of major vessels (the number of visible vessels on coronary angiography), chest pain type, thalassemia status (thal), ST-segment depression after exercise/drug (oldpeak), exercise-induced angina (exang), and peak heart rate (max hr). The partial dependence curve further revealed stable patterns such as the monotonic increase of oldpeak, the step-by-step increase of num major vessels, and the reverse effect of max hr (31–33).

### 4.2 Mechanistic

A 2x upscaling achieves relatively favorable model prediction performance. For small sample sizes, moderate local smoothing and neighborhood synthesis can reduce estimation variance and stabilize the decision boundary. When upscaling exceeds 2x, the slight “stretching” of the true distribution by the synthesized samples accumulates, introducing distribution drift and amplifying high-dimensional noise, leading to a decline in test set performance (34).

RF naturally mitigates high variance and multiple correlation issues through out-of-bag bootstrapping and feature subsampling. Its splitting criterion is insensitive to scale changes and outliers, and it can robustly capture nonlinearities and high-order interactions in mixed (continuous + categorical) features. Compared to boosted trees and neural networks, RF is more likely to strike a balance between bias and variance given the current sample size and feature dimensionality, and offers a direct advantage in interpretability (Permutation/Gini + SHAP).

Combining SHAP and PDP reveals that the model’s discriminative information is driven by a small number of key clinical factors, which interact with each other. ST-segment depression (oldpeak) increases approximately monotonically with predicted risk, with a slope increasing at approximately 1.0–1.5, quantitatively reproducing the dose–response relationship of “lower pressure, higher risk” for subendocardial ischemia during stress testing. The number of major coronary vessels affected (num major vessels) exhibits a step-wise gain, with greater involvement associated with higher risk, consistent with the dose–response relationship between anatomical stress and adverse outcomes. Exercise-induced angina (exang) and chest pain type (chest pain type) demonstrate significant stratified positive contributions. Highly typical/easily provocative symptom profiles provide a strong signal of near-term event risk under stress conditions, consistent with clinical triage experience. In contrast, peak heart rate (max hr) has an overall negative contribution. High heart rate capacity corresponds to better exercise tolerance and myocardial reserve, and is therefore protective. Its marginal effect shifts from a decline to a plateau around 140 beats/min, suggesting the existence of a possible physiological threshold. In addition, thal (perfusion/ischemic lesion-related stratification) has a sustained upward impact on risk, supplementing the functional information of oldpeak/exang. Overall, the model integrates the three-dimensional evidence chain of “structure (vascular involvement) - function (ST segment, perfusion) - stress response (symptoms, heart rate reserve)” into a consistent risk narrative with quantitative marginal contributions, which not only explains the global ranking but also supports individual-level predictions (33, 35, 36).

### 4.3 Comparison with previous work

While previous work primarily evaluated classification models using discriminant metrics such as AUC, this study first modeled outcomes as continuous risk scores, assessed fit quality using MAE/RMSE/R^2^, and then connected the results to the actual discriminant and confusion matrices through thresholding, thereby building a bridge between “risk quantification and threshold decision-making” that is closer to the clinical process. Compared to common oversampling methods such as SMOTE, our feature space moving average synthesis emphasizes local continuity and medically reasonable domain constraints, improving stability while maintaining marginal/conditional distributions. This is particularly evident when amplified by 2x (37).

### 4.4 Clinical significance

From a clinical perspective, this study provides three areas of translational value. First, based on the thresholding results of RF, a high-sensitivity threshold can be selected in screening/triage scenarios to reduce missed diagnoses. In resource-constrained or pre-invasive testing settings, a balanced threshold (recommended in this study) can be used to balance sensitivity and specificity. Second, SHAP and PDP decompose model outputs into traceable clinical factors (such as ST-segment depression, exercise-induced angina, number of major vessels/type of chest pain), facilitating communication with patients about risk factors and providing quantitative evidence for determining who requires further testing/intensive follow-up. Third, RF strikes a good balance between training and inference costs, stability, and interpretability, making it suitable as a “secondary decision maker” for initial screening in outpatient and emergency departments or inpatient settings, and can be run on existing structured EHR data. From a clinical application perspective, the proposed model should be regarded as a supplementary risk stratification tool rather than a replacement for established clinical risk scores. The newly added ROC-AUC, PR-AUC, calibration analysis, Brier score, and Youden index-based thresholded metrics provide additional information on discrimination, probability reliability, and threshold-based decision performance. These results support the potential use of the model in preliminary screening or triage scenarios based on routinely available structured clinical variables.

### 4.5 Limitations

Our study has several limitations. The limited sample size of a single public dataset makes it difficult to fully capture complex clinical heterogeneity and may limit the external generalizability of the proposed models across different clinical populations and healthcare settings. Second, the augmentation method relies on local smoothing and neighborhood assumptions, which may lead to conservative estimates near the true boundary of samples. Although the moving-average-based augmentation was constrained within clinically plausible values to reduce unrealistic or biased synthetic samples, a direct comparison with standard oversampling techniques such as SMOTE was not performed in this study and will be considered in future work. Although nested cross-validation and training-only augmentation strategies were adopted to reduce overfitting and data leakage risks, some models still exhibited partial train-validation performance gaps, particularly under higher augmentation conditions. In addition, PCA was used primarily as an exploratory linear visualization approach and may not fully capture nonlinear relationships among clinical variables or provide direct physiological validation of the projected components. Third, the choice of threshold still depends on the cost function of the specific scenario and requires collaborative calibration with clinical teams in real-world workflows. Direct comparison with established clinical risk scores and formal decision curve analysis were not performed because the public dataset lacked several variables and longitudinal outcome information required for standard clinical scoring and net-benefit evaluation. Future work will incorporate multicenter external cohorts, follow-up time information, and continuous imaging/biochemical time series, and introduce utility-sensitive learning/decision curve analysis to further align clinical costs.

## 5 Conclusion

In cardiovascular data, moderate data augmentation (2×) significantly improved model fit quality and discriminant stability. In a multi-model comparison, random forests achieved the favorable balance between accuracy, robustness, and interpretability. SHAP and PDP analyses revealed a risk-driven spectrum centered on ST-segment depression, exercise-induced angina, number of major coronary arteries, chest pain type, and peak heart rate. Individual-level contribution decomposition supported risk stratification and thresholding decisions. These results provide a methodological basis and practical path for deploying interpretable and implementable cardiovascular risk prediction tools in real-world settings. Subsequent multicenter external validation and scenario-based cost function optimization will advance the model from research to clinical practice.

## Data Availability

The original contributions presented in the study are included in the article/Supplementary material, further inquiries can be directed to the corresponding author/s.
