# Supplementary material for: Enhancing clinically cardiovascular machine learning model for risk prediction via sample augmentation
Source: Front Med (Lausanne). 2026 Jun 9;13:1849464. doi: 10.3389/fmed.2026.1849464 (PMC13286745; doi:10.3389/fmed.2026.1849464)
Supplement: Supplementary file 1 [file Table_1.DOCX]

Supplementary Material

# Supplementary Tables

# Supplementary Table 1. Hyperparameter configurations of the evaluated machine learning models used in this study.

| **Model** | **Hyperparameters** | |
| --- | --- | --- |
| **SVR** | C | 4 |
|  | degree | 8 |
|  | epsilon | 0.014 |
|  | gamma | 2 |
|  | kernel | rbf |
| **MLP** | activation | relu |
|  | batch_size | 32 |
|  | hidden_layer_sizes | 64,128,128 |
|  | learning_rate_init | 0.007 |
|  | max_iter | 1500 |
|  | early_stopping | TRUE |
|  | n_iter_no_change | 50 |
| **RF** | n_estimators | 418 |
|  | max_depth | 11 |
|  | min_samples_leaf | 2 |
|  | min_samples_split | 3 |
|  | bootstrap | FALSE |
|  | max_features | sqrt |
| **XGBoost** | n_estimators | 459 |
|  | learning_rate | 0.117 |
|  | max_depth | 7 |
|  | colsample_bytree | 0.87 |
|  | subsample | 0.89 |
|  | random_state | 42 |
|  | min_child_weight | 7 |
| **LightGBM** | n_estimators | 390 |
|  | learning_rate | 0.122 |
|  | max_depth | 7 |
|  | num_leaves | 7 |
|  | colsample_bytree | 0.84 |
|  | subsample | 0.8 |
|  | random_state | 42 |
|  | min_child_samples | 5 |

# Supplementary Table 2. Comprehensive performance of different augmentation factors and models based on MAE, RMSE, and R^2^, reported as mean ± standard deviation.

| **Amplification factor** | **Algorithm** | **Training set** | | | **Test set** | | |
| --- | --- | --- | --- | --- | --- | --- | --- |
|  |  | **R^2^** | **MAE** | **RMSE** | **R^2^** | **MAE** | **RMSE** |
| **0** | **SVR** | 0.874 ± 0.033 | 0.074 ± 0.004 | 0.146 ± 0.007 | 0.567 ± 0.029 | 0.213 ± 0.009 | 0.328 ± 0.012 |
|  | **MLP** | 0.872 ± 0.031 | 0.111 ± 0.005 | 0.178 ± 0.005 | 0.542 ± 0.027 | 0.217 ± 0.007 | 0.336 ± 0.010 |
|  | **RF** | 0.857 ± 0.034 | 0.135 ± 0.005 | 0.188 ± 0.003 | 0.574 ± 0.031 | 0.251 ± 0.009 | 0.325 ± 0.021 |
|  | **XGBoost** | 0.864 ± 0.039 | 0.119 ± 0.003 | 0.193 ± 0.004 | 0.559 ± 0.027 | 0.225 ±0.008 | 0.315 ± 0.019 |
|  | **LightGBM** | 0.852 ± 0.030 | 0.128 ± 0.004 | 0.191 ± 0.005 | 0.516 ± 0.020 | 0.215 ± 0.004 | 0.334 ± 0.017 |
| **1** | **SVR** | 0.927 ± 0.042 | 0.047 ± 0.021 | 0.116 ± 0.002 | 0.619 ± 0.031 | 0.202 ± 0.003 | 0.306 ± 0.016 |
|  | **MLP** | 0.908 ± 0.046 | 0.089 ± 0.023 | 0.148 ± 0.006 | 0.611 ± 0.030 | 0.183 ± 0.006 | 0.302 ± 0.014 |
|  | **RF** | 0.989 ± 0.047 | 0.029 ± 0.001 | 0.051 ± 0.003 | 0.631 ± 0.033 | 0.208 ± 0.002 | 0.302 ± 0.012 |
|  | **XGBoost** | 0.921 ± 0.043 | 0.089 ± 0.002 | 0.141 ± 0.002 | 0.586 ± 0.028 | 0.214 ± 0.003 | 0.321 ± 0.013 |
|  | **LightGBM** | 0.908 ± 0.039 | 0.106 ± 0.005 | 0.157 ± 0.003 | 0.589 ± 0.027 | 0.212 ± 0.005 | 0.319 ± 0.010 |
| **2** | **SVR** | 0.981 ± 0.048 | 0.022 ± 0.001 | 0.068 ± 0.001 | 0.778 ± 0.035 | 0.133 ± 0.007 | 0.241 ± 0.011 |
|  | **MLP** | 0.956 ± 0.038 | 0.048 ± 0.002 | 0.144 ± 0.006 | 0.741 ± 0.036 | 0.145 ± 0.003 | 0.291 ± 0.013 |
|  | **RF** | 0.991 ± 0.045 | 0.021 ± 0.001 | 0.047 ± 0.002 | 0.789 ± 0.033 | 0.129 ± 0.006 | 0.226 ± 0.009 |
|  | **XGBoost** | 0.976 ± 0.047 | 0.044 ± 0.003 | 0.077 ± 0.002 | 0.765 ± 0.040 | 0.131 ± 0.005 | 0.239 ± 0.010 |
|  | **LightGBM** | 0.959 ± 0.050 | 0.072 ± 0.009 | 0.111 ± 0.004 | 0.749 ± 0.039 | 0.154 ± 0.006 | 0.247 ± 0.019 |
| **3** | **SVR** | 0.975 ± 0.051 | 0.028 ± 0.007 | 0.078 ± 0.003 | 0.749 ± 0.028 | 0.177 ± 0.004 | 0.253 ± 0.011 |
|  | **MLP** | 0.945 ± 0.048 | 0.062 ±0.008 | 0.121 ± 0.005 | 0.735 ± 0.029 | 0.133 ± 0.009 | 0.255 ± 0.012 |
|  | **RF** | 0.982 ± 0.040 | 0.034 ± 0.006 | 0.066 ± 0.004 | 0.771 ± 0.030 | 0.141 ± 0.008 | 0.232 ± 0.010 |
|  | **XGBoost** | 0.972 ± 0.037 | 0.051 ± 0.005 | 0.082 ± 0.002 | 0.743 ± 0.032 | 0.153 ± 0.007 | 0.253 ± 0.012 |
|  | **LightGBM** | 0.951 ± 0.042 | 0.081 ± 0.003 | 0.121 ± 0.003 | 0.725 ± 0.027 | 0.171 ± 0.008 | 0.261 ± 0.010 |

# Supplementary Table 3. Discrimination, calibration, and thresholded classification performance of the evaluated models on the validation set.

|  | **Youden index** | **ROC-AUC** | **PR-AUC** | **brier score** | **accuracy** | **Sensitivity** | **Specificity** | **PPV** | **NPV** | **F1-score** | **F2-score** |
| --- | --- | --- | --- | --- | --- | --- | --- | --- | --- | --- | --- |
| **SVR** | 0.5508 | 0.980041378 | 0.983977149 | 0.049118129 | 0.950549451 | 0.949494949 | 0.951807229 | 0.959184 | 0.94047619 | 0.954314721 | 0.951417004 |
| **MLP** | 0.3881 | 0.959230863 | 0.945905362 | 0.058755292 | 0.923076923 | 0.95959596 | 0.879518072 | 0.904762 | 0.948051948 | 0.931372549 | 0.948103792 |
| **RF** | 0.5465 | 0.988682001 | 0.990704696 | 0.046178906 | 0.961538462 | 0.96969697 | 0.951807229 | 0.96 | 0.963414634 | 0.964824121 | 0.967741935 |
| **XGBoost** | 0.5103 | 0.983448947 | 0.985085645 | 0.0486276 | 0.961538462 | 0.96969697 | 0.951807229 | 0.96 | 0.963414634 | 0.964824121 | 0.967741935 |
| **LightGBM** | 0.5914 | 0.984544238 | 0.98669566 | 0.05165987 | 0.934065934 | 0.909090909 | 0.963855422 | 0.967742 | 0.898876404 | 0.9375 | 0.920245399 |
